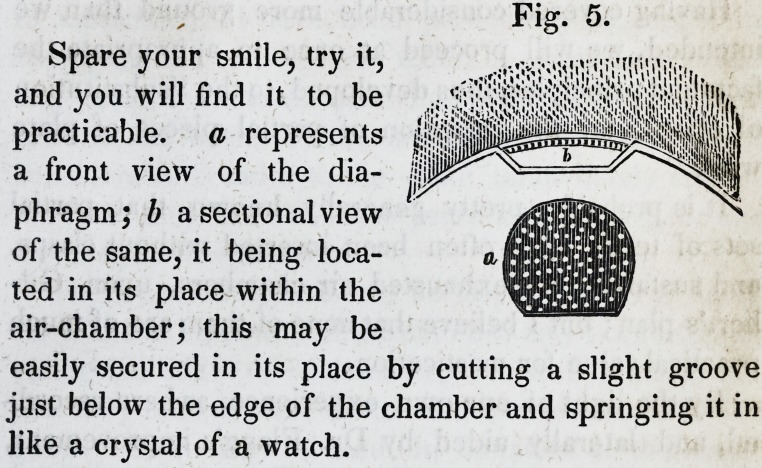# Flagg on Lateral Cavity Plates

**Published:** 1850-01

**Authors:** J. F. B. Flagg

**Affiliations:** Surgeon Dentist.


### ARTICLE VI.

[From the Dental News Letter.]

### FLAGG *on Lateral Cavity Plates.*

MESSRS. JONES, WHITE & CO.

*Gentlemen:*—The operation for adjusting an entire upper set of teeth to the mouth, is one of great delicacy, and often presents difficulties the most experienced in our art are not altogether prepared to meet. I have reference to those cases that depend entirely upon atmospheric pressure for their adhesion.

The purpose of this communication is to recommend to the profession a plan which I have recently introduced in my practice, with the most satisfactory results; and the purpose of the invention is the more perfectly to secure to the upper jaw artificial teeth, when recourse is had to atmospheric pressure; to prevent the rocking or canting of such teeth, when antagonized by the under teeth in mastication, and to restore the upper jaw to its original fulness, so desirable to retain a natural tone of voice.

I am aware that "cavity suction plates" have been more or less used for many years, of various construction, with different degrees of success; and that one has more recently obtained letters patent, under the name of "central cavity plates," for the greater adhesion to the roof of the mouth; but these have required that much metallic substance should be carried over the entire bony palate, conflicting with the sense of taste, and having its *chamber* so located as to cause a protuberance in the mouth, entirely at variance with all anatomical formation; inducing changes from the natural tone of voice, difficulty of articulation, and other serious complaints from many who have resorted to them.

The nature of my invention consists in so forming the plate, upon which the artificial teeth are secured, as to perfectly fit the jaw in all its parts, as at present ordinarily practiced, except in that portion of the dental ridge immediately behind, and in line with the grinding or molar teeth. At this point I recommend that the plate be made sufficiently depressed to have no bearing upon the jaw, thus forming *lateral cavities* or chambers, which, when exhausted of the air by suction, secures the whole plate firmly in its proper position. This depression of the plate, also, restores to the jaw that fulness which it had lost by absorption, consequent upon the extraction of teeth.

The alteration which I make in the plate, is accomplished in the following manner: after obtaining an accurate impression of the jaw, in wax, I cut out a portion of the wax along the line of the grinding teeth upon each side of the mould, about one inch in length by three-eighths in width, and one-tenth in depth, of an oval and cup-like form; taking care not to warp or otherwise alter the general character of the mould. Into this wax mould I cast my plaster of paris; this plaster cast, when sufficiently *set* or hardened, I remove from the wax, and trim with a knife suitable to prepare the necessary metallic casts for striking up the plate. Should the plate not fit the jaw perfectly from this impression, I recommend that a similar plate be made of *sheet lead;* adjust this lead plate to the jaw, *taking care not to derange the suction cavities;* and, by placing this lead carefully into the former wax mould, cast the plaster once more into it, and proceed as before.

I remain, truly yours,

J. F. B. FLAGG, M. D., *Surgeon Dentist.*

---

REMARKS BY THE CAZENOVIA EDITOR.—The subject of the excellent article by Dr. P. H. Austen, in the July number of the Journal, has long engrossed oru particular attention, as being one of the most "eminent practical importance," and we are delighted at the prospect of its general discussion and elucidation.

The doctor has most emphatically "struck a vein"—not that it was undiscovered or unworked before, but that he has directed the public opinion of our fraternity to the subject in such a manner, and followed it up with such a suit of earnest interrogations which appeal so decidedly to our professional perfection, that it cannot fail of soon developing its largest resources.

In his article "*on the Use of Clasps for the Retention of Artificial Teeth,*" he inquires "how far may the principle of Cleveland's air cavity be substituted for clasps in the retention of partial pieces of plate work?" After discussing the subject in a manner which cannot fail to interest every one who reads it, he closes with an invitation to the profession to detail the result of their various discoveries and experience in the premises; with a view to establish and perfect a system for a general application of the principle.

We most willingly respond to the call, and take the earliest opportunity of placing before the readers of the Journal, the result of our own experience, as well as that of others, as we understand them, so far as they have a bearing upon the subject at issue.

We shall consider, *First.* The atmospheric principle as applied to the support of dental plates.

*Secondly.* Wherein the principle has been heretofore but imperfectly applied.

*Thirdly.* The various efforts made to perfect the application of this principle. And,

*Lastly.* An appropriation of all of the facts and principles as developed in the foregoing, so far as is practicable, to "the substitution of clasps for the retention of partial pieces of plate work."

Perhaps some of our readers will imagine that we are assuming, unnecessarily, broad ground, compared with the actual requisitions of our subject. We differ with them, considering it as we do, indispensable to the end we have in view, while, at the same time, we assure them that a two-fold advantage arises from it. First, by giving a full review of the various methods of applying the atmospheric principle in full sets, known up to this time, we may be so fortunate as to throw some new light upon this increasingly valuable branch of mechanical dentistry; while having covered this ample ground, we can, secondly, the more clearly and easily elucidate our last proposition, based as it is, upon the general facts then established.

"*Of the Atmospheric Principle as applied to Plates.*"

It is, perhaps, sufficient for us to say, that plates de signed to cover a considerable portion of the palatine arch, are struck from accurate impressions of the mouth in such a manner as to be well adapted to the gums, when, on withdrawing the air from between, the plates adhere to the gums with a greater or less degree of tenacity, which, however, is always increased by time.

"*This principle has heretofore been but imperfectly applied,*" because, from the very nature of the construction of the plates, it is impossible to derive any thing more than a limited degree of the advantages of the atmospheric principle, for the reason, that when a plate, no matter how well adapted to them, is applied to the gums, and an effort is made to exhaust the air from beneath, the gums along the line, and behind the edge of the plate, are drawn down so as to meet it, forming powerful, and impassable valves, to the utter resistance of any effort from without to withdraw the air from the central part of the plate; so that in nine cases out of ten, the atmospheric principle does not act upon a breadth of surface of more than three or four lines around the edge of the plate, and this, at a point where the suction is easily and constantly broken, while the whole *central* portion of the plate, which is comparatively protected, and would be least liable to disturbance, is left in a comparative normal state.

The unsteadiness and rocking of some plates, while others which are feebly sustained, on being carefully drawn downwards seem to *fall*, as it were, one or two lines, and then adhere with surprising tenacity—plates which resist a wonderful degree of downward force, but drop on the slightest lateral pressure, these and others are but striking illustrations of the fact that there is, in a majority of atmospheric sets, but little or no vacuum in the central part of the plate, where, the least reflection teaches us, it is most needed, so long as permanence and solidity of adaptation are ends for which we strive. But from the foregoing, we see that from the very force of circumstances, the central part at best, is the last and least to feel the atmospheric influence; which leads us very naturally, as it almost suggests, to our third proposition—"*The various efforts to perfect the application of this principle.*" As we have already professed to have bestowed some considerable attention to this subject, we trust we shall be pardoned if we introduce some of our own experience in advance of others, to whom we will soon refer, at the same time giving them the largest credit.

In the winter of 1845, a gentleman applied to us for a double set of teeth. Upon examining his mouth, we were surprised to find that every vestige of the alveolar ridge was absorbed from the superior maxillary, leaving it diminutive, and almost entirely flat, while the muscles of the mouth sprung immediately from its scarcely defined borders. The inferior maxillary was fully equal to ordinary presentations. Here was a case for the atmospheric principle! for we had long since foresworn springs, and, to increase our perplexity, our patient informed us with a degree of confidence, positively shocking, that he had no doubt but that with our assistance, he would be a great proficient in the masticating way, before the month was out.

We took a plaster impression in the usual manner, then cast the model and swage, and finally fitted a very thick plate to the latter, in the most perfect manner—indeed, throughout the whole process, we took unusual pains, anticipating as we did, trouble in the end. In this last, we were not disappointed, for though it fitted most admirably at every point, the slightest muscular movement of the mouth would throw it down from its position. It was in vain that we pared down the edges of the plate to avoid its contact with the lateral muscles—indeed, some of these so far infringed upon the surface of the palate, as almost to assume the character of the rugæ, so that cutting away the plate to meet their demands was wholly impracticable. To sum up the matter, we were eminently successful in our *failure!* We sought our rest that night deeply impressed with this last fact; but with "*nil desperandum*" in our heart, we went to philosophising upon the principle of atmospheric pressure. All that we have transcribed under the head of our second proposition, occurred to us at that time, and for a while, forgetting the realm of toothdom, we went abroad, investigating this principle in general, as exemplified in mechanics, the arts and sciences, &c. This led us by remembered lectures, and readings in natural philosophy, and visions of water pumps with their valves, and air pumps with their receivers, which strong men could not remove, came before our eyes. The heavy laden cars of the atmospheric railway hurried past our vision with a swiftness corresponding to their own movements, and then came up the vacuo-making feet of all gravity-contradicting insects, who traverse our ceilings without fear of a fall. The blood sucker, too, gave in his instalment towards embodying an idea, illustrating, that like an assiduous shoemaker, he would stick to the *last!* Then came in parenthesis, the thought of human blood suckers, those Shylocks who apply the *suck-in* principle when they wish to make a *draw!* And then, again, we gave a glance quite far back to the days of our childhood, when in sport, we played with the "suck-leather," and lifted, thereby, large stones, little deeming that we did it under the patronage of any philosophical principle, and long before we knew that nature ever got up so much *feeling*, as to "abhor a vacuum" or any thing else. Here, at this primitive application of the atmospheric principle, we arrested our mental wanderings, to inquire into the *modus operandi* by which the suction of a circular piece of leather will lift a stone of a thickness equal to its own diameter, and near three times that in breadth.

A thick piece of spongy leather is selected, and cut into a circle of six or eight inches in diameter, a hole is then punctured in the centre large enough to admit a small strong cord, at one end of which, a knot is tied; the cord is passed through the leather and drawn up firmly, so that the knot is imbedded into it; the whole is then soaked in water for a short time—the valvular outward-opening pores of the skin thus being rendered elastic—when it is ready for use.

The leather is pressed firmly down upon the surface of a stone, and then the little operator, who has studied no book but the unwritten one of experience and observation, who never heard of a theory, until after it was *practiced*, gradually begins to draw up on the cord which is attached to the centre; the leather following it, rises in the form of a cone, leaving a vacuum underneath. Our little hero watches the progress of affairs and finds that a very tenacious alliance is already being formed between these uncongenial substances; he perceives, too, that immediately around the border of the elevation, and extending out towards its edge, the leather is apparently pressed down with exceeding firmness, and that just in proportion to the perfection of the vacuum in the centre, does the leather press down upon the stone and fortify the tenacity already established;—a little more patience, a long pull, and a strong pull, and the victim stone is *hanging* by a cord, with a cap upon its head, amid the triumphant crowing of our juvenile *ketch!*

Now suppose a similar piece of leather with several strings attached—some reaching out to near its border, should be pressed down upon the stone in the same manner as before, then let an equally distributed force be applied to all of them in a direction upwards, no matter how carefully it is done, one would be separated from the other with scarce an effort.

Again, apply as before, then draw up the centre string, and form a vacuum there; upon drawing equally upon the strings as above, the central one included, the stone will be raised from the ground, as at first. Indeed, the central vacuum being established, according to the extent of the surface unappropriated, you can form lesser vacuums all around it.

Next morning, we took our case of failure again in hand. Applying a coat of wax to our plaster model, the thickness of about an eighth of an inch in the centre, we let it gradually diminish in depth until it reached within about four or five lines of the edge all around. With the model thus prepared, we made another suit of swages, and struck up our plate anew. On applying the plate to the mouth of our patient, and directing him to exhaust the air from beneath it, we found it to adhere with a considerable degree of tenacity. But this much was no new discovery, for about the same thing had been practiced by dentists oftentimes before, to prevent plates from riding on the hard parts of the mouth—especially when impressions were taken in wax. We then, with an exceeding fine drill, made a small hole in the central part of the cavity thus formed, and afterwards reamed it out to a cone shape, the *base* terminating outwards, and not being much more than half a line in diameter; then taking a piece of gold nearly pure, with a small jewelers' bow lathe, we filed it down so as nearly to fit, and close up the conical hole in the plate, leaving an extension of the same to come up through the plate, in the form of a stem. Taking this little *valve*—for it will soon be entitled to the name—from the lathe, we passed it through the plate, with it thus attached, we applied it again to the lathe, the stem answering for one of the bearings as before. Now, with some fine scrapings of a Scotch hone, mixed with oil, we ground the conical valve to the cone-shaped hole until it was air-tight, we then cut off the valve at its base so as to leave it even with the plate. Having previously rolled down a piece of gold to exceeding thinness, we cut out a piece of it in the form of a cross, at the center where the angles met, we punctured a hole to receive the stem of the valve; bending the four extremities downwards equally; we formed what artizans would call a spider-leg spring. Now, the valve being in its place, we placed the spring immediately over it, on the upper surface of the plate, and pressed it down—the stem of the valve passing through the whole in the center of the spring, until it reached within about an half a line of the plate; with a small pair of forceps, we then seized the stem above the spring, and by pressure, flattened it down, so as to form a kind of a head, thus detaining it attached, and holding it to its place. We have subsequently used a spring made of a single strip of gold, with one end attached to the plate, like a tongue to an accordeon, making the orifice in it for the reception of the stem in the form of a slit, to give play to the valve, in adapting itself to the hole in the plate. It is an improvement, too, to solder on a piece of plate where the orifice in the chamber is to be made so as to have it of double thickness at that point.

Fig. 1, represents an enlarged view of the valve and socket without the spring; *a* represents the plate in which the conical valve is fitted; *b* is the stem, and *c* represents the base of the valve; *d* represents the valve, spring and plate combined, being of about natural size.

Applying the plate again to the mouth of our patient, we directed him to exhaust the air from beneath. In doing which, the valve is drawn down from its place, giving the air an opportunity to pass out; the moment the force of suction ceases, that instant the valve passes up to its place, thus preventing ingress of air from without, and preserving the vacuum at each step of its formation. At the first effort of suction, the plate adhered to the gums with great tenacity; after one or two more efforts, it adhered with such force that we were only able to remove it by forcing a small flat probe between the plate and gums. Here we had an air-pump, complete in all its parts, and perfect in all its operations, giving the patient the ability to remove a part, or all, of the air, at his pleasure, and of producing every degree of graduation from a partial to a complete vacuum. And yet, the whole apparatus is so simple in its arrangement, that the slightest care will insure it against injury. In addition to this, the principle of suction has full play upon all parts of the plate as before. We completed our case with the most gratifying results, and it is worn to this day, equally fulfilling all of the offices performed by any double set of teeth within our knowledge.

It will naturally be supposed that we made many experiments to ascertain the best practicable form to give the chamber between the plate and gums. In some plates we swaged down a prolonged cone, or cup-shaped cavity, somewhat in imitation of the metallic bells, and glass receivers, connected with an ordinary air-pump. This form, especially, if it had a well defined and abrupt edge, had the advantage that it would keep its place with less atmospheric force than any other, for the reason, not only that the gums around the whole border of the cavity came down as a valve and effectually prevented the air from coming in from without, but also, that in coming down, it takes a position at right angles with the plate, and gives it, perhaps, aside from atmospheric pressure, as great a degree of solidity and permanence as though it was swaged over a hard protuberance, at that point.

Our original form had the advantage of forming no perceptible chamber or obstruction in the mouth to interfere with articulation, and the free movement of the tongue, but owing to its border being undefined, it required, to give it the necessary permanence, a force of suction much greater than the other form. This, in some mouths, would be a serious objection. Indeed, air chambers must ever be more or less objectionable with some patients, especially those with spongy and tender gums, and those of scrofulous habit. For this, we have a remedy, of which we will soon speak.

At the next meeting of our society, we laid the result of our experiments thus far, before several of our brethren, at the same time requesting them to join with us in further perfecting the application of the principle. The summer following, while at New York,

Dr. J. Parmly showed a full set of teeth of his construction, upon our plan. To the case exhibited to us, he originally designed to attach springs, but dispensed with them, as he found them unnecessary, as he thought they ever would be thereafter, so gratifying had been the result of his experience in the application of the valve and chamber. At this time, Dr. J. B. Rich, suggested to us a sharp pointed drill, with a broad shoulder, for insuring the hole in the plate always of one form; and also a steel plate with a corresponding conical hole, in which to form the valve. Dr. Parmly also gave us several very excellent hints in regard to locating the chamber so as to balance the lateral and opposing forces of the teeth; the principle being to locate the chamber at a point where it will balance its opposing forces with the most certainty.

During the operation of cupping a friend of ours along the line of the sciatic nerve, for a severe inflammation of that organ; after applying two cups, on reaching for a third, we found our assortment literally *broken*, and reduced to an old one which we had never used, for the reason that its edges were so sharp that it seemed cruelty to do so. Here was a dilemma, the scarificator had done its work—right skilfully had those well-martialed rows of lancets (lancers) gone their *round;* it was a pity to lose it all, so we clapped on the rejected cup to save what we had done! To our surprise, notwithstanding, the cup drew well—far better than the others, it did not draw the flesh but a very little within the cup; it was exceedingly firm in its place, and not at all painful. This we found to be the case on every repetition of the experiment; and what seemed still more singular was the fact that while the skin over which the blunt edged cups were used, remained discolored for a long time, those parts over which the sharp edged cup had been placed, almost immediately became smooth and colorless. The rationale of the matter lies here, the sharp-edged cup imbeds itself readily and firmly into the surface of the flesh, so that immediately on the vacuum being formed, the flesh around the border of the cup, lies at right angles with, and against its edge, firmly holding the parts to their place, and leaving those within free to act, and be acted upon. While with the blunt-edged cup, the flesh is drawn far within, the blood-vessels are soon strangulated, and the parts congested. Ah, thought we, the old rejected cup has a destiny to fulfil after all, and it has done it.

We immediately commenced making an application of the hint to the formation of our air chambers, and soon found that, the sharper the edge the better.

When we form a chamber, we always cast two leaden counter-swages; with the first, we swage up the plate as far as we can, then while the plate is on the zinc cast, with a small instrument and a mallet, we drive the plate to its place all around the border of the elevated part over which the chamber is formed, then afterwards, finish with the remaining counter-swage.

Plates fitted in this manner, adhered with such a degree of tenacity, that in most cases, we found we could dispense with the valve. The next time we communicated with Dr. Parmly, we found he had also dispensed with its use in most cases.

In April, 1848, we first saw one of Gilbert's central cavity plates, and was surprised to find it but slightly differing from our own, without the valve; the chief difference consisted in ours having a better defined edge, and the walls of the chamber being more at right angles with the plate.

We have no doubt but that Mr. Gilbert invented his central cavity plate, while at the same time, we are no more in doubt about inventing our own. We made ours with the valve, known to a few of our friends, in 1845. His, without the valve, became publicly known some two years after; we have already expressed, in the Journal, that we believe in a coincidence of *discovery;* perhaps, as Mr. Gilbert's *chamber* was first *publicly* known, he is entitled to the whole credit; we shall set up no claims over him, we only care to benefit the profession in every way that in our power lies, and at this particular writing, we only care to answer the question of our friend, Dr. Austen, and trust, that we shall soon arrive at a point, where we can do so, to our liking.

During the month of August, 1848, we had the pleasure of traveling from New York to Philadelphia, with our excellent friend, Dr. J. A. Cleaveland, of Charleston, S. C. At that time, we compared notes on the subject of our air-chambers; a description of his is already published in the Journal; it is exceedingly ingenious, and has several advantages over other forms. Dr. C. is no doubt the inventor of the vacuo-chamber that bears his name, and is entitled to great credit for the same; he, in conjunction, or, rather, about the same time with myself, and perhaps others, has seized upon a principle, and applied it in his own original way.

On the next page will be found, a sectional view of the various methods to which we have referred. In our drawing, we have inadvertently given too much depth to the chambers.

Fig. 2.

Fig. 4.

Fig. 3.

Fig. 2, represents that of Dr. Cleaveland's. *a* represents a section of the gums; *b*, the plate; *c*, the covering of plate which forms the base, or flooring of the chamber, and *d*, the chamber. Fig. 3, represents a plate with such a chamber as we have before described, without a valve. Fig. 4, represents one with a valve.

With Dr. Cleaveland's improvement, we are always insured a good adaptation, a well defined edge at the point of union of the plate with the gums, and what is of great importance, the chamber is constructed in such a manner as to present a smooth and unbroken surface beneath it, so as not to interfere with any of the legitimate operations of the organs of the mouth. We have lately improved ours in this respect, by soldering on a ring of gold plate, so as to have it bridged across from the lower edge of the chamber to the plate, as represented by the dotted lines in Figs. 3 and 4.

In remarking that air-chambers must ever be more or less objectionable to patients with spongy gums, we intimated that we had a remedy. Take a piece of thin gold plate, and file it into the shape of the chamber at its opening, then perforate it full of small holes with a punch, as thickly together as the material will admit of; with this cover over the chamber, making it serve as a diaphragm, as represented in Fig. 5.

Fig. 5.

Spare your smile, try it, and you will find it to be practicable. *a* represents a front view of the diaphragm; *b* a sectional view of the same, it being located in its place within the air-chamber; this may be easily secured in its place by cutting a slight groove just below the edge of the chamber and springing it in like a crystal of a watch.

Heretofore we have located the chamber in various parts of the mouth, in partial, as well as in whole sets; our success with partial sets without clasps, however, never gave us much satisfaction, especially where teeth were to be located at opposite sides of the mouth, and without the valve—except in one instance, indeed, this had one clasp, and we never felt satisfied in the matter in all respects, until we read Dr. Flagg's excellent article on the subject of *lateral cavities*, which will be found in the present number of the Journal. Since reading it, we have constructed three upper sets on his plan with the most pleasing results. We once inserted a partial set of teeth, by placing a clasp on one side, and a lateral cavity on the other, and that plate has successfully withstood severe tests of mastication ever since; why we did not think to substitute a lateral cavity for the clasp, we cannot tell; *we did not*, and Dr. F. has now shown us how *easy* a thing it is to do it.

We always take our impressions in plaster, and form the chambers, by building wax upon the model, in the form we design to give them.

Having covered considerable more ground than we intended, we will proceed at once to appropriate the facts and principles, thus developed, to the "substitution of clasps, for the retention of partial pieces of plate work."

It is probably pretty generally known that partial sets of teeth have often been inserted without clasps, and sustained by exhausted air-chambers, upon Gilbert's plan; but I believe that none of them are of much practical value for mastication.

By the light of our own experience, as here recorded, and laterally aided by Dr. Flagg's improvement, which we have fully tested, we will now venture to respond to Dr. Austen's inquiry, by affirming, that in a large majority of cases, partial sets of teeth may be inserted, of *practical value*, without the aid of clasps, and upon the general principle of Cleaveland's improvement, by constructing two opposing lateral cavities in the plate, care being taken to give them well defined edges, as described above. To insure complete success in all cases, it is only necessary to insert a valve in the chambers. With the chamber alone, you are only enabled to exhaust a part of the air, for reasons which we have already given; the partial vacuum formed, however, is sufficient for general success. But, by the aid of the valve you may exhaust every particle of the air if you choose. The valve also gives you the advantage of using less plate, and of constructing a smaller chamber.

Lateral cavities, in many instances, may be partially concealed by extending them under the teeth upon the plate, as in full sets. The immediate front teeth, from one to four, can be successfully inserted in this manner easier than others, for the reason that they are less used in mastication. I have been particularly successful in supplying one or two front teeth, by forming a broad and shallow chamber upon the plate, extending back towards the center of the mouth, taking care to give considerable breadth to the plate immediately behind the remaining front teeth, to serve as a bearing, or antagonist, to the chamber.

The lateral chambers may be disposed of in as many ways as the ingenuity of the operator may suggest, providing the edges of such cavities are well defined, and that they always come down to the true surface of the gums. They may be made in imitation of the rugæ, extending along on either side of the mouth, only taking care to have the apparent subdivisions of each communicated.

Our article has extended much beyond the length we intended to give it; we did not expect at the outto be very brief on so extensive a subject, but after all, our random pen has taken large liberties with us; still, we think, as every fact herein recorded has proven useful to us in arriving at the end we had in view, they may be useful to others, to other ends. We sincerely hope, though we are among the first to answer Dr. Austen's question, that there may be many between ourselves and the last, and that we shall soon have the combined experience of many.

Will some of our readers give us their experience with lateral cavities, in lower sets of teeth?

## Figures and Tables

**Fig. 1 f1:**
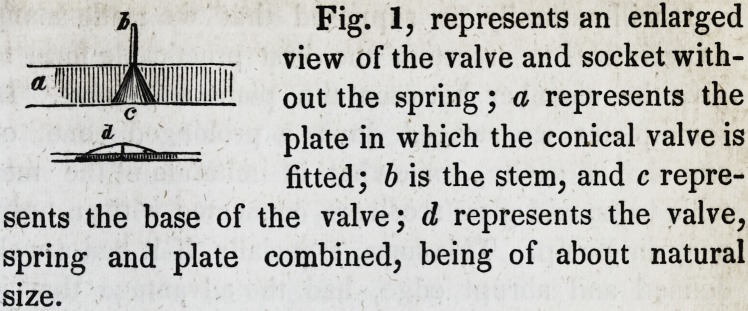


**Figure f2:**
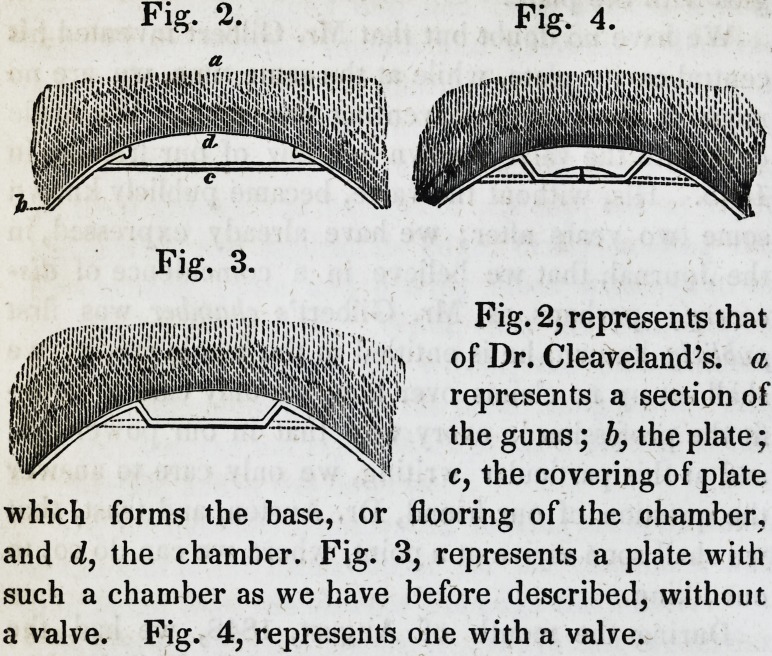


**Fig. 5. f3:**